# Contribution of Processes in SN Electrodes to the Transport Properties of SN-N-NS Josephson Junctions

**DOI:** 10.3390/nano13121873

**Published:** 2023-06-16

**Authors:** Vsevolod Ruzhickiy, Sergey Bakurskiy, Mikhail Kupriyanov, Nikolay Klenov, Igor Soloviev, Vasily Stolyarov, Alexander Golubov

**Affiliations:** 1 Skobeltsyn Institute of Nuclear Physics, Lomonosov Moscow State University, 119991 Moscow, Russia; 2 Dukhov All-Russia Research Institute of Automatics, 101000 Moscow, Russia; 3 National University of Science and Technology MISIS, 119049 Moscow, Russia; 4 Faculty of Physics, Moscow State University, 119991 Moscow, Russia; 5 Center for Advanced Mesoscience and Nanotechnology, Moscow Institute of Physics and Technology, 141700 Dolgoprudny, Russia; 6 Faculty of Science and Technology, MESA+ Institute for Nanotechnology, University of Twente, 7500 AE Enschede, The Netherlands

**Keywords:** proximity effect, SNS Josephson junctions, current-phase relationship

## Abstract

In this paper, we present a theoretical study of electronic transport in planar Josephson Superconductor–Normal Metal–Superconductor (SN-N-NS) bridges with arbitrary transparency of the SN interfaces. We formulate and solve the two-dimensional problem of finding the spatial distribution of the supercurrent in the SN electrodes. This allows us to determine the scale of the weak coupling region in the SN-N-NS bridges, i.e., to describe this structure as a serial connection between the Josephson contact and the linear inductance of the current-carrying electrodes. We show that the presence of a two-dimensional spatial current distribution in the SN electrodes leads to a modification of the current–phase relation and the critical current magnitude of the bridges. In particular, the critical current decreases as the overlap area of the SN parts of the electrodes decreases. We show that this is accompanied by a transformation of the SN-N-NS structure from an SNS-type weak link to a double-barrier SINIS contact. In addition, we find the range of interface transparency in order to optimise device performance. The features we have discovered should have a significant impact on the operation of small-scale superconducting electronic devices, and should be taken into account in their design.

## 1. Introduction

**Figure 1:**
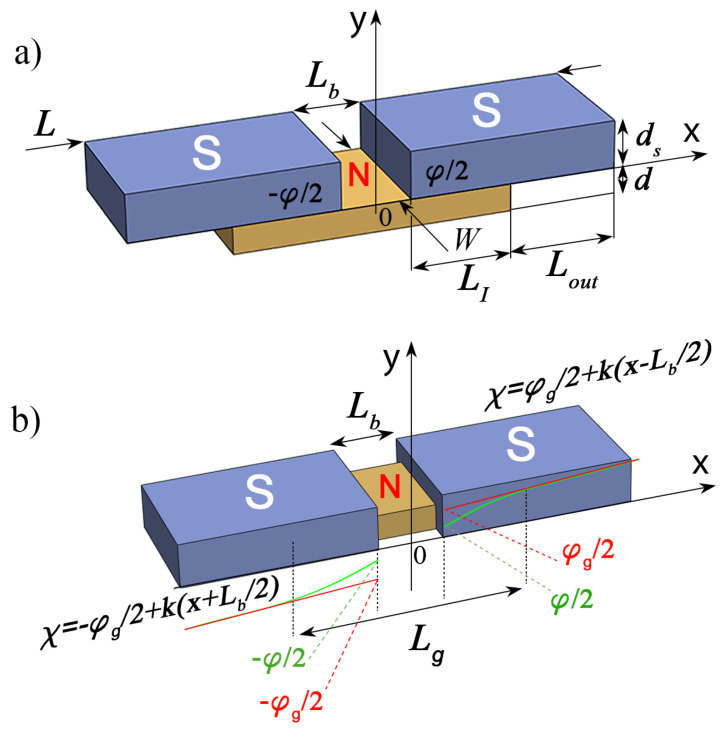
(**a**) Sketch of Josephson SN-N-NS bridge and (**b**) sketch of Josephson SNS bridge. Here, $L_{b}$ is the distance between the S electrodes, *W* is the width of the structure, $L_{I}$ is the length of the SN interface, $d_{s}$ and *d* are the thickness of the S and N films, respectively, φ is the order parameter phase difference across the junctions, $\varphi_{gl}$ is the global phase difference across the junction, and χ(x) is the asymptotic coordinate dependence of the electrode order parameter phase away from the weak link region.

Recently, there has been renewed interest in studying planar Josephson structures with strong supercurrent concentration near the weak junction. Figure 1 shows the most common configurations of such structures. These are bridges consisting of superconducting electrodes (S) either in contact with a sublayer of normal (N) metal (Figure 1a) or simply connected by a metal bridge (Figure 1b). The structures shown in Figure 1a are called SN-N-NS bridges. The structures in Figure 1b are called variable thickness bridges (VTB) or constant thickness bridges (CTB), depending on whether the thickness of the bridge film *d* is equal to the thickness of the S-electrodes $d_{s}$ or significantly less, respectively.

These structures are now widely used in the fabrication of nano-SQUIDs [1,2,3,4,5,6,7,8,9,10,11] as well as in the design and implementation of digital circuits [12,13,14]. The ordinary metals Nb, NbN [3,4,5,12,15], TiN [8,9,9], Al [2,16,17], Pb [18], Au [19,20,21], and Cu [22] as well as two-dimensional electron gas [23,24] and topological insulators [25,26,27,28,29,30,31,32,33,34,35,36,37,38,39,40,41,42,43,44,45,46] have been used as the weak coupling material of the Josephson elements in these devices. The calculations performed in [47,48,49] indicated the prospect of using SN-N-NS bridges as basic elements of digital and analogue superconducting devices [50].

One of the most important characteristics of the bridges is the relationship between the superconducting current *I* flowing through them and the phase difference φ of the order parameters of their superconducting (S) electrodes [51,52]. The question of what the value φ means and how to determine it correctly depends both on the geometry of the Josephson structure and on the transport properties of its boundaries.

In this paper, we solve this question regarding SN-N-NS Josephson bridges. It is important to note that the developed approach can be applied to any structure in which there is a concentration of supercurrentin S-electrodes in the vicinity of their boundary with the weak coupling region.

## 2. Correct Determination of φ as Measured by Experiment

The problem of correctly defining φ has been conventionally solved in the simplest model, the so-called Rigid Boundary Conditions (RBC), to describe the properties of the S-electrodes. The RBC model has been the workhorse model most often used in the analysis of processes in Josephson junctions [53,54,55,56,57,58]. It is assumed that all nonlinear and non-equilibrium processes in Josephson structures are localized in the weak coupling region between two two superconducting (S) electrodes (see Figure 1b). Reverse effects of these processes on electrode superconductivity are considered to be negligible. The electrodes are in a stationary and equilibrium state, so that the order parameter modules $\Delta_{0}$ and the anomalous Green functions characterizing their superconducting state are independent of the spatial coordinates and coincide with their equilibrium values calculated for a solitary superconductor. In this model, the setting of a bias current through the Josephson contact is provided by a χ linearly increasing with the coordinate phase of the order parameter $\Delta =\Delta_{0}\exp\left\{i\chi \right\}$, which experiences a jump at the geometric boundary of the weak coupling region by the value φ (the red line in Figure 1b). This value is often called the Josephson phase difference at contact [51].

Further research allowed us to formulate the areas of applicability of this model for the case of almost equal thickness and width of the N and S films. It has been demonstrated [59,60,61,62,63,64,65,66] that suppression of superconductivity in S electrodes due to the proximity effect and the effect of depairing of superconducting correlations by bias supercurrent leads to a mismatch between the phases of Green’s function and the order parameter at the NS boundaries, which no longer coincide with each other. For this reason, it is impossible to introduce a physically meaningful Josephson phase difference φ between the NS interfaces.

The suppression of superconducting correlations in the vicinity of the interfaces of S electrodes with the material of the weak coupling region actually means the spatial expansion of this region. It is no longer determined by the geometric boundaries separating the materials of the structure, so that the Josephson phase difference of the order parameters φ becomes not a measurable quantity and in no way characterizes the properties of the Josephson structure. The weak coupling region expands up to the values of $L_{g}$. This length defines the positions of the regions inside the S electrodes, outside of which the modules of both $\Delta_{0}$ and the anomalous Green’s functions reach values independent of spatial coordinates and their phases coincide with each other and increase linearly with the growth of the coordinate *x* along the *x*-axis. Extrapolation of this linear dependence to the geometric boundaries of materials determines the global Josephson phase difference $\varphi_{g}$. The introduction of $\varphi_{g}$ allows us to consider the Josephson contact as a serial connection of the structure with the current–phase relation $I(\varphi_{g})$ and the inductance of the S electrodes. All effects initiated by delocalization of the weak link region (the green line in Figure 1) are taken into account in the shape of the $I(\varphi_{g})$ dependence. This provides a junction description convenient for designing and studying processes in devices having such delocalised Josephson contacts.

In [67], it was concluded that the desired parameters of the Josephson contact $I_{c}∼0.1$ mA, $I_{c}R_{N}∼0.8$ mV can be achieved at $L_{b}∼50$ nm, W∼40 nm. These scales are quite comfortable for modern methods in the CMOS industry. The problem is to obtain such $I_{c}$ and $I_{c}R_{N}$ with accuracy 2%, and these lengths should be implemented with an accuracy up to 1 nm. Because implementation of this accuracy in the bridge-type planar technologies used for superconducting VLSI circuits would require very advanced CMOS nodes, the authors of [67] concluded that this type of junction can be used only in devices in which the parameter spreads are not important.

In fact, such a rigorous conclusion is rather a consequence of the specific geometry of bridge shown in Figure 1b. Recently [47], it was demonstrated that in the bridge geometry shown in Figure 1a there are no such strict restrictions on *W*, $L_{b}$, and *d*. This is due to the delocalization of the weak region due to the finite transparency of the SN boundaries.

In the microscopic theory of superconductivity [68,69], the presence of a finite transparency of the interface between a massive superconductor and a thin non-superconducting metal is characterized by a suppression parameter $\gamma_{BM}=\gamma_{B}d/\xi_{n},$ $\gamma_{B}=\left(R\xi_{s}\right)/\left(\rho_{n}\xi_{n}\right)$. Here, *R* is the specific boundary resistance, $\xi_{s}$ is the coherence length of the S electrodes, and $\rho_{n}$ and $\xi_{n}$ are the resistivity and decay length, respectively, of the weak link material. Typical values of the suppression parameter $\gamma_{BM}=1÷3$ at interfaces between Nb and a thin Al film were obtained by comparing theoretical predictions [69] with experimental results [70,71,72] obtained in NbAl-AlO_*x*_-Nb and NbAl-AlO_*x*_-AlNb tunnel structures. The existence of a finite interface transparency between two normal metals was confirmed experimentally in [73,74,75,76,77,78].

In an SN-N-NS bridge, the finite interface transparency leads to violation of the rigid boundary conditions used in the above estimates [67] and delocalization of the weak coupling region [47] (see Figure 1a). In the limit $L_{b}≲\xi_{n}$, both the critical current $I_{c}$ and the $I_{c}R_{N}$ product are mainly determined in these junctions by the suppression parameter $\gamma_{BM}$ rather than by the bridge geometrical factors $L_{b}$ and *W*. There is no need for additional structuring of the width of the bridge film [47]. Its width may coincide with the width of the composite electrode, and may be determined by the requirements for the line width of a technological process. In MIT Lincoln Laboratory technology, the lower and upper limits on *W* (150 nm ≲W≲250 nm) are determined by the requirement of current carrying capacity and uniformity of the bias current distribution across the width of the electrodes, respectively [79]. There are no strict requirements for the reproducibility of the gap between the electrodes formulated in [67]. Due to the delocalization of processes in the weak link region, its effective size along the direction of the current significantly exceeds $L_{b}$. Moreover, numerical calculations show that in SN-N-NS junctions at $L_{b}≲\xi$ and $\gamma_{BM}≳1$, the critical current is practically independent on $L_{b}$.

However, it should be noted that the conclusions formulated in [47] were obtained under the assumption that the phase of the electrode order parameter at the SN boundaries χ(x,0)=±φ/2=const does not depend on the coordinate *x*. Obviously, this assumption is too strong. It does not take into account the heterogeneous nature of the current supply from the N-film to the S-electrode, and leads to a violation of the current conservation law (divJ=0) in composite SN-electrodes. Thus, even in the absence of superconductivity suppression in the S part of a composite SN electrode, χ(x,0) should depend on *x* due to the mutual nature of the proximity effect and the effect of current suppression of superconductivity, and cannot be used as an argument in the current-phase relation. The solution to this problem is to formulate and solve the two-dimensional problem of finding the spatial distribution of the supercurrent in the SN electrodes, determining $\varphi_{g}$, and finding the $I(\varphi_{g})$ relation. This is exactly the purpose of the present work.

## 3. Model

We consider a normal (N) metal film connecting two massive superconducting (S) electrodes of length $L_{I}+L_{out}$ located at a distance ±L/2 from the center of the film (see Figure 1a). Here, $L_{I}$ is the length of overlap between the S and N layers, while $L_{out}$ is the length of the free outer part of the S electrode. We place the origin of coordinates on the upper surface of the N film in the middle of the SN-N-NS structure and direct the *x* and *y* axes along and perpendicular to the SN interfaces, respectively. The existence of the proximity effect between the N and S materials should lead to the induction of superconducting correlations into the N metal, leading to Josephson coupling between the S banks.

We suppose that the dirty limit condition is satisfied for all the metals, the critical temperature of the weak link material is equal to zero, and its thickness *d* is much smaller than the decay length $\xi_{n}={\left(D/2\pi T_{c}\right)}^{1/2}$. Here, D, is the diffusion coefficient of the weak link material and $T_{c}$ is the critical temperature of the S electrode. In the normal metals commonly used in technology, such as Cu, Au, and Al, the coherence length is $\xi_{n}$ 50–100 nm (see [78,80] and references therein). Thus, for N layers with a thickness of *d* 5–10 nm, it is easy to realize a strong inequality between *d* and $\xi_{n}$. Due to the symmetry of the problem, we may consider it for positive *x* only.

The conditions

(1) $$\;d\ll \xi_{n},\;\gamma_{m}=\frac{\rho_{n}\xi_{n}}{\rho_{s}\xi_{s}}\frac{d}{\xi_{n}}\ll 1$$

permit us to neglect the suppression of superconductivity in the S film due to proximity effect and reduce the two-dimensional Usadel equations [81] in the N film to a one-dimensional problem [47,49]:

(2) $$\xi_{eff}^{2}\frac{\partial }{\partial x}\left(G^{2}\frac{\partial \Phi }{\partial x}\right)-\Phi =-\delta \exp\left\{i\chi \left(x,0\right)\right\},$$

$$L_{b}/2\leq x\leq \left(L_{I}+L_{b}\right)/2$$

(3) $$\xi_{eff}^{2}=\frac{\gamma_{BM}}{G\left(G_{s}+\gamma_{BM}\omega \right)},\;\delta =\frac{G_{s}\Delta }{\left(G_{s}+\gamma_{BM}\omega \right)}.$$

(4) $$\frac{\partial }{\partial x}\left(G^{2}\frac{\partial \Phi }{\partial x}\right)-\omega G\Phi =0,\;0\leq x\leq L_{b}/2$$

Here, Φ and $G=\omega /{\left(\omega^{2}+\Phi \Phi^{*}\right)}^{-1/2}$ are Usadel Green’s functions, $\omega =\left(2n+1\right)T/T_{C}$ are Matsubara frequencies normalized on $\pi T_{C}$, the coordinate *x* is normalized on $\xi_{n}$, the modulus of the order parameter in the S electrode Δ is normalized on $\pi T_{C}$, and $\rho_{s}$ and $\xi_{s}$ are the resistivity and coherence length of the S film, respectively.

Due to (1), we assume that the suppression of superconductivity in the S electrodes is negligible and that the value of the critical junction current $I_{c}$ is significantly less than the preparation current of the S films. Under these conditions from the Usadel equation in the S electrodes, it follows that in this approximation the phases of the order parameter Δ(x,y) and anomalous Usadel Green’s functions $\Phi_{s}\left(x,y\right)$ coincide with each other, while their modules equal their equilibrium values in a superconductor at a given temperature *T*:

(5) $$\Phi_{s}\left(x,y\right)=\Delta \left(x,y\right)=\Delta_{0}\exp\left\{\chi \left(x,y\right)\right\},\;G_{s}=\frac{\omega }{\sqrt{\omega^{2}+\Delta_{0}^{2}}}.$$

The phase χ(x,y) obeys the Laplace equation

(6) $$\frac{\partial^{2}}{\partial x^{2}}\chi +\frac{\partial^{2}}{\partial y^{2}}\chi =0.$$

It is convenient to normalize the *x* and *y* coordinates in (6) as well as all of the geometrical lengths $d_{n},$ $d_{s},$ $L_{b},$ $L_{I},$ $L_{out}$ on $\xi_{n}.$ Equations (2), (4) and (6) must be supplemented by the boundary conditions.

At $x=L_{b}/2$, they are determined from the requirement of continuity of the Φ functions and their first derivatives:

(7) $$\frac{\partial \Phi (L_{b}/2+0)}{\partial x}=\frac{\partial \Phi (L_{b}/2-0)}{\partial x},$$

$$\Phi \left(\frac{L_{b}}{2}+0\right)=\Phi \left(\frac{L_{b}}{2}-0\right).$$

At $x=L_{b}/2+L_{I}$,

(8) $$\frac{\partial }{\partial x}\Phi =0,$$

and at x=0,

(9) ∂∂xReΦ=0,ImΦ=0

where ReΦ and ImΦ are the real and imaginary parts of Φ, respectively.

At $x=L_{b}/2$, $0\leq y\leq d_{s}$, $y=d_{s},$ $L_{b}/2\leq x\leq L/2$, and y=0, $L_{b}/2+L_{I}\leq x\leq L/2$, which follow from the demand of absence of any supercurrent across the interfaces

(10) $$\frac{\partial }{\partial x}\chi \left(\frac{L_{b}}{2},y\right)=0,\;\frac{\partial }{\partial y}\chi \left(x,d_{s}\right)=0,\;\frac{\partial }{\partial y}\chi \left(x,0\right)=0.$$

We further assume that the characteristic size of the S electrodes $\left(L-L_{b}\right)/2$ significantly exceeds the characteristic lengths ($\xi_{n},\xi_{eff}$) at which a spatial redistribution of the supercurrent density $J_{s}$

(11) $$J_{s}\left(x,y\right)=\left\{J_{sx},J_{sy}\right\}=S_{1}\nabla \chi \left(x,y\right),\;$$

$$S_{1}=\frac{2\pi T}{e\rho_{s}\xi_{n}}\sum_{\omega >0}^{\infty }\frac{G_{s}^{2}\Delta_{0}^{2}}{\omega^{2}}$$

in the S electrodes is possible. Under this condition, at x=L/2, $0\leq y\leq d_{s}$ there is a uniform distribution of the superconducting current density $J_{s}$ over the thickness of the S and N films, that is, $J_{y}=0$ and

(12) $$J_{sx}=S_{1}\frac{d}{dx}\chi \left(L_{g}/2,y\right)=\frac{I}{d_{s}},$$

where *I* is a full supercurrent flow across the junction. Expression (12) determines the boundary condition at x=L/2.

(13) $$\frac{d}{dx}\chi \left(L/2,y\right)=\frac{I}{S_{1}d_{s}},\;0\leq y\leq d_{s}.$$

The last boundary condition at y=0, $L_{b}/2\leq x\leq L_{b}/2+L_{I}/2$ follows from the current conservation low as well. It reads that decay of supercurrent $I_{n}$ in the weak link film at a point *x* located under the S electrode should be equal to the density of supercurrent injected at this point into this S electrode:

(14) $$S_{1}\frac{d}{dy}\chi \left(x,0\right)=\frac{d}{dx}I_{n},$$

(15) In(x)I0=TTc∑ω≥0∞G2ω2ImΦ∂ReΦ∂x−ReΦ∂ImΦ∂x,

where $I_{0}=J_{0}Wd$, $J_{0}=2\pi T_{c}/e\xi_{n}\rho_{n}$, and *W* is the width of the structure.

The boundary value problem formulated above was solved by numerical methods. It is naturally divided into two interrelated tasks. The first consists of calculating the distribution of the supercurrent (15) along the normal film, that is, in solving the boundary value problem (2), (4), (7)–(10) for a given phase distribution χ(x,0). The second task is to use the finite element method to find the dependencies of χ(x,y) and $J_{s}\left(x,y\right)$ by solving the Laplace Equation (6) with the boundary conditions (10), (13), (14). The solution of the Laplace equation χ(x,y) is determined up to a constant. We chose its value from the condition $\chi (L_{b}/2,0)=\varphi /2$.

Calculation of current characteristics can be accomplished using the iterative method for a given phase φ. At the zeroth iteration, a calculation is made for a constant value of the superconducting phase χ(x,0)=φ/2=const along the S electrode. The obtained value of the total current *I* and the derivative of the current $dI_{n}/dx$ along the electrode are used as the boundary conditions for solving the problem for the S electrode. In the next step, we substitute the resulting distribution χ(x,0) into the Usadel’s Equations (2) and (4). Then, the iterative cycle is repeated until the value of the critical current stops changing. With the values of the parameters under consideration, four iterative cycles are sufficient for this. Extrapolation of the linear dependence χ(x,0) obtained in the region x≈L/2 on the boundary of the composite SN electrode with bridge film $\left(x=L_{b}/2\right)$ determines $\varphi_{g}$, and allows us to find the desired dependence $I(\varphi_{g})$.

**Figure 2:**
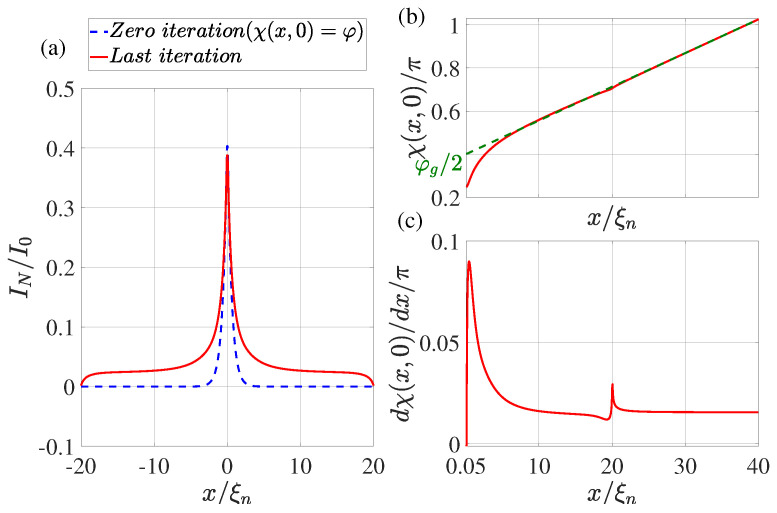
(**a**) The coordinate dependence of the current $I_{n}$ flowing through the N film. The dotted blue line shows the result obtained at the zeroth iteration (χ(x,0)=φ), while the solid red line shows the dependence obtained at the last iteration. (**b**) Distribution of phase of the superconducting order parameter χ(x,0) at the last iteration. The dotted green line shows the linear extrapolation of the χ(x,0) function from the region x≤L/2. (**c**) The derivative of the phase of the order parameter as a function of coordinate *x* at the last iteration. Calculations have been performed for $T/T_{c}=0.5$, $L_{I}/\xi_{n}=20$, φ=π/2, $d_{n}/\xi_{n}=0.1$, $d_{s}/\xi_{n}=10$, L=80, $\rho_{s}/\rho_{n}=10$, $\gamma_{BM}=0.5$, $L_{b}/\xi_{n}=0.1$.

**Figure 3:**
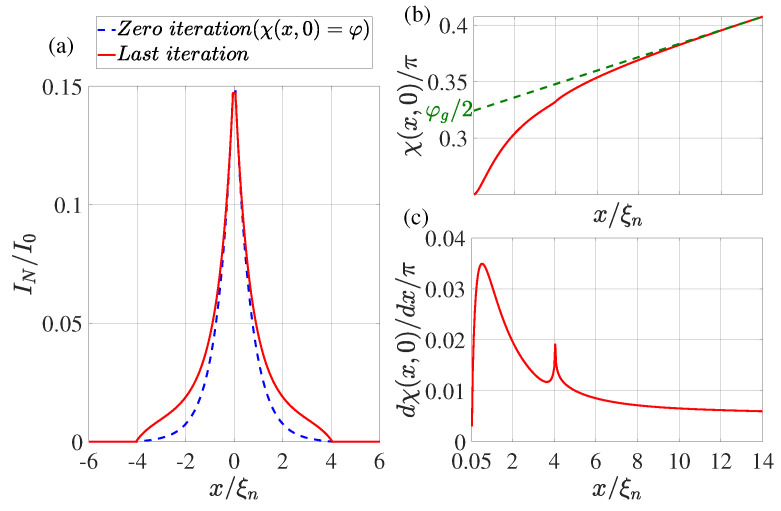
(**a**) The coordinate dependence of the current $I_{n}$ flowing through the N film. The dotted blue line shows the result obtained at the zeroth iteration (χ(x,0)=φ), while the solid red line shows the dependence obtained at the last iteration. (**b**) Distribution of phase of the superconducting order parameter χ(x,0) at the last iteration. The dotted green line shows the linear extrapolation of the function χ(x,0) from the region x≤L/2. (**c**) The derivative of the phase of the order parameter as a function of coordinate *x* at the last iteration. Calculations have been performed for $T/T_{c}=0.5$, $L_{I}/\xi_{n}=4$, φ=π/2, $d_{n}/\xi_{n}=0.1$, $d_{s}/\xi_{n}=10$, L=28, $\rho_{s}/\rho_{n}=10$, $\gamma_{BM}=1$, $L_{b}/\xi_{n}=0.1$.

**Figure 4:**
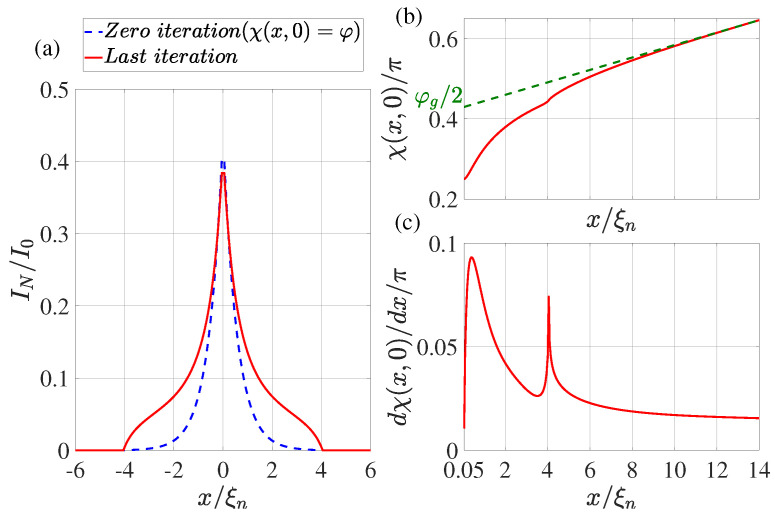
(**a**) The coordinate dependence of the current $I_{n}$ flowing through the N film. The dotted blue line shows the result obtained at the zeroth iteration (χ(x,0)=φ), while the solid red line shows the dependence obtained at the last iteration. (**b**) Distribution of phase of the superconducting order parameter χ(x,0) at the last iteration. The dotted green line shows the linear extrapolation of the function χ(x,0) from the region x≤L/2. (**c**) The derivative of the phase of the order parameter as a function of coordinate *x* at the last iteration. Calculations have been performed for $T/T_{c}=0.5$, $L_{I}/\xi_{n}=4$, φ=π/2, $d_{n}/\xi_{n}=0.1$, $d_{s}/\xi_{n}=10$, L=28, $\rho_{s}/\rho_{n}=10$, $\gamma_{BM}=0.5$, $L_{b}/\xi_{n}=0.1$.

**Figure 5:**
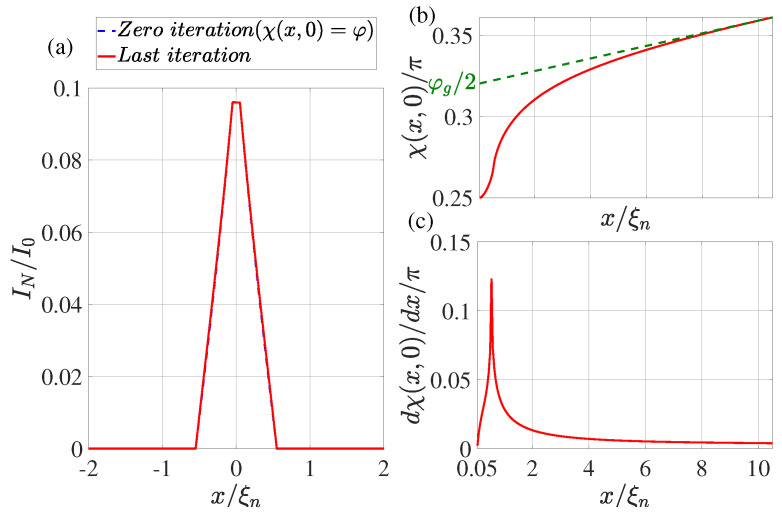
(**a**) The coordinate dependence of the current $I_{n}$ flowing through the N film. The dotted blue line shows the result obtained at the zeroth iteration (χ(x,0)=φ), while the solid red line shows the dependence obtained at the last iteration. (**b**) Distribution of phase of the superconducting order parameter χ(x,0) at the last iteration. The dotted green line shows the linear extrapolation of the function χ(x,0) from the region x≤L/2. (**c**) The derivative of the phase of the order parameter as a function of coordinate *x* at the last iteration. Calculations have been performed for $T/T_{c}=0.5$, $L_{I}/\xi_{n}=0.5$, φ=π/2, $d_{n}/\xi_{n}=0.1$, $d_{s}/\xi_{n}=10$, L=20, $\rho_{s}/\rho_{n}=10$, $\gamma_{BM}=1$, $L_{b}/\xi_{n}=0.1$.

In the practical implementation of the calculation method described above, we limited ourselves to the following set of parameters: $T/T_{c}=0.5$, φ=π/2, $d_{n}/\xi_{n}=0.1$, $d_{s}/\xi_{n}=10$, $L_{out}=10$, $\rho_{s}/\rho_{n}=10$, $L_{b}/\xi_{n}=0.1$. We performed the calculation for two values: $\gamma_{BM}=1,0.5$ and $L_{I}/\xi_{n}=20,4,0.5$. The calculation results are shown in Figure 2, Figure 3, Figure 4 and Figure 5.

## 4. Supercurrent Distribution in the N Part of SN Electrodes

The dotted blue line in Figure 2a, Figure 3a, Figure 4a and Figure 5a shows the supercurrent distribution in the N part of SN electrodes at the initial step of iteration. It can be seen that at $L_{I}/\xi_{n}=20$ (see Figure 2a) in this step the current completely outflows from the N layer into the S film at a characteristic length of $\xi_{eff}$. Note that it is this current distribution that was used earlier in [47] when calculating the parameters of SN-N-NS bridges. The iterative solution of the problem presented in Figure 2a by the solid red line shows that the area in which the currents are redistributed between the N and S films is expanding. Moreover, part $I_{n}$ of the full current continues to flow along the N layer even away from the bridge, reaching a value that is weakly dependent on *x*. There is practically no leakage of current from the N to the S part of the SN electrode in this area. This component of the current finally leaves the N layer in the vicinity of $x=L_{I}/2.$ The area in which $I_{n}$ weakly depends on *x* shrinks with decreasing $L_{I}$. Thus, with $L_{I}/\xi_{n}=4$ (see Figure 3a and Figure 4a), the difference in the dependencies of $I_{n}\left(x\right)$ at the initial (solid blue line) and final (dotted red line) iteration stages is no longer so significant. The smaller it is, the larger the parameter $\gamma_{BM}$, that is, the larger $\xi_{eff}$. Finally, for $L_{I}<\xi_{eff}$, the current component $I_{n}$ turns out to be independent of *x*. In this case, the current is injected into the S film through a small area without changing the phase of the order parameter along the SN boundary, meaning that χ(x,0)=φ. This is confirmed by the numerical calculations for $L_{I}/\xi_{n}=0.5$ presented in Figure 5a.

## 5. Spatial Dependence of the Order Parameter Phase χ(x,0) along the SN Interface

It is convenient to analyze the nature of current leakage from the N film into the S electrode by examining the coordinate dependencies of the phase of the superconductor order parameter along the SN boundary shown in Figure 2b,c, Figure 3b,c, Figure 4b,c and Figure 5b,c. It can be seen that regardless of the values of $L_{I}$ in the vicinity of $x=L_{b}/2$ there is a sharp increase in the derivatives dχ(x,0)/dx to values several times larger than their values in the region of $x≲L_{I}$. After reaching the maximum, the derivatives decrease, experiencing a singularity at $x=L_{b}/2+L_{I}$ due to the final leakage of current into the S electrode.

This behavior of χ(x,0) indicates a significant concentration of $J_{sx}$ components of the current density in the vicinity of $x≳L_{b}/2$.

**Figure 6:**
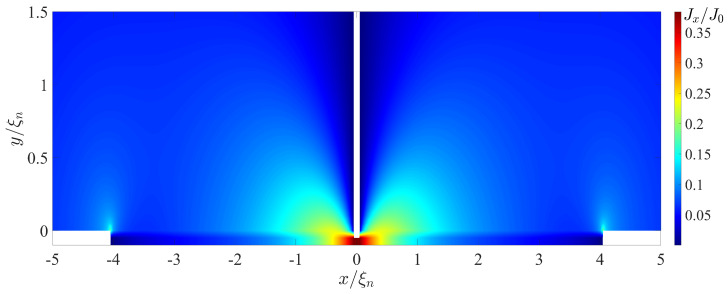
Coordinate dependence of the $J_{x}\left(x,y\right)$ component of the supercurrent density calculated for $T/T_{c}=0.5$, $L_{I}/\xi_{n}=4$, φ=π/2, $d_{n}/\xi_{n}=0.1$, $d_{s}/\xi_{n}=10$, L=28, $\rho_{s}/\rho_{n}=10$, $\gamma_{BM}=0.5$, $L_{b}/\xi_{n}=0.1$.

**Figure 7:**
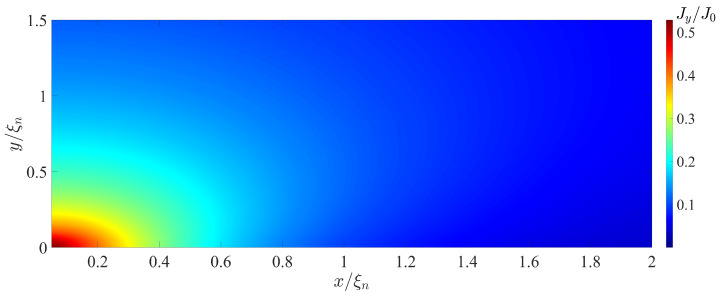
Coordinate dependence of the $J_{y}\left(x,y\right)$ component of the supercurrent density calculated for $T/T_{c}=0.5$, $L_{I}/\xi_{n}=4$, φ=π/2, $d_{n}/\xi_{n}=0.1$, $d_{s}/\xi_{n}=10$, L=28, $\rho_{s}/\rho_{n}=10$, $\gamma_{BM}=0.5$, $L_{b}/\xi_{n}=0.1$.

Figure 6 and Figure 7 show spatial distributions of $J_{x}\left(x,y\right)$ and $J_{y}\left(x,y\right)$ supercurrent components in the SN-N-NS bridge calculated for $L_{I}/\xi_{n}=4$ and φ=π/2. These spatial distributions clearly demonstrate that in the region $L_{b}/2≲x≲L_{b}/2+L_{jx}$ with $L_{jx}\approx 1.5\xi_{n}$, the concentration of the current component $J_{x}$ really occurs in an area whose thickness is about 4 times less than $d_{s}$. The characteristic scale $L_{jy}$ of the injection area of the current component $J_{y}$ into the S electrode is approximately equal to $0.6\xi_{n}$. With $\gamma_{BM}=0.5$, this is completely consistent with the theoretical estimate of the size of this region $\xi_{n}{\left(\gamma_{BM}\right)}^{1/2}$ in [47].

It should be noted that the difference between $L_{jx}$ and $L_{jy}$ is due to the fact that $L_{jy}$ is determined by the value $\xi_{eff}$ at ω=πT, while $L_{jx}$ plays the role of the Josephson penetration depth and is inversely proportional to the square root of the local value of critical current density of the SN interface.

The concentration of the current component $J_{x}$ leads to an increase in the local inductance value $L_{loc}$ per unit length of this region, which is inversely proportional to the characteristic scale of the current concentration region. This scale turns out to be significantly less than $d_{s}$. To extract the additional contribution in $L_{loc}$ from the magnitude of the linear inductance of the S-electrode $L_{lin}$, we must determine the magnitude of $\varphi_{g}$:

(16) $$\frac{\varphi_{g}}{2}=\chi \left(\frac{L}{2},0\right)-\frac{d\chi (L/2,0)}{dx}\left(\frac{\left(L-L_{b}\right)}{2}\right)$$

as shown in Figure 2b, Figure 3b, Figure 4b and Figure 5b. The dotted line in Figure 2b, Figure 3b, Figure 4b and Figure 5b shows the asymptotic dependence of χ(x,0) for large values of *x*, which determines the value of $\varphi_{g}/2$ for $x=L_{b}/2$. This definition of $\varphi_{g}$ opens up the possibility of representing the SN-N-NS structure in the form of two bundled elements connected in series, namely, the Josephson junction with CPR $I(\varphi_{g})$ and the linear inductance $L_{lin}$ of the S film.

Such an equivalent scheme of Josephson contact actually implies taking into account part of the inductance of the S electrodes in the form of changing the shape of contact current–phase relation. With this description, the part of the S electrodes ($L_{b}/2\leq x\leq L_{gl}/2$) in which there is a noticeable deviation of the dependence of χ(x,0) from the linear one formally turns out to belong to the weak coupling region. The calculation results presented in Figure 2b, Figure 3b, Figure 4b and Figure 5b allow us to conclude that $L_{gl}\approx 5\xi_{n}$ and weakly depend on $L_{I}$ and $\gamma_{BM}$.

It is interesting to note that a completely opposite procedure was proposed earlier in the RBC model [82]. In [82], the authors suggested describing a Josephson contact with non-sinusoidal dependence I(φ) as a series connection of inductance and contact with a sinusoidal of the I(φ) relation.

## 6. Current–Phase Relation $I_{s}\left(\varphi_{\mathrm{gl}}\right)$

**Figure 8:**
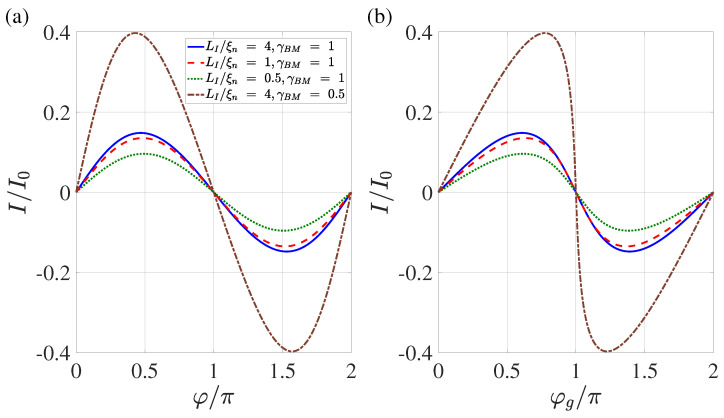
Current–phase relation I(φ) (**a**) and dependence of current on the global phase $I(\varphi_{g})$ (**b**) calculated for four combinations of $L_{I}$ and $\gamma_{BM}$. These combinations are ($L_{I}/\xi_{n}=4,\gamma_{BM}=1$), ($L_{I}/\xi_{n}=1,\gamma_{BM}=1$), ($L_{I}/\xi_{n}=0.5,\gamma_{BM}=1$), and ($L_{I}/\xi_{n}=4,\gamma_{BM}=0.5$). Other parameters are $T/T_{c}=0.5$, $d_{n}/\xi_{n}=0.1$, $d_{s}/\xi_{n}=10$, L=20, $\rho_{s}/\rho_{n}=10$, $L_{b}/\xi_{n}=0.1$.

Our numerical calculations show that the shape of the current–phase relation I(φ) for $T/T_{c}=0.5$ and $L_{I}/\xi_{n}≳2$ (see Figure 8) differs slightly from the one calculated earlier in [47]. It is close to a sinusoidal shape; however, unlike the results obtained in [47], the maximum in I(φ) is slightly shifted not to the area of φ>π/2, but towards φ<π/2. Such deformation is typical for Josephson structures, as the superconductivity of their electrodes is suppressed by the current flowing through them [59,61,63]. This indicates that in SN-N-NS bridges in the region $L_{b}/2\leq x≲L_{b}/2+\xi_{n}$ there is a slight suppression of the superconductivity induced into the N film by the current injected into it.

In contrast, the deviation of $I_{s}\left(\varphi_{g}\right)$ from the sinusoidal shape turns out to be significant (see Figure 8a). It can be seen that the critical current $I_{c}$ achieved at $\varphi_{g}=\varphi_{m}>\pi /2$, and that the parameters $I_{c}$ and $\varphi_{m}$ are both dependent on $L_{I}$ and $\gamma_{BM}$.

**Figure 9:**
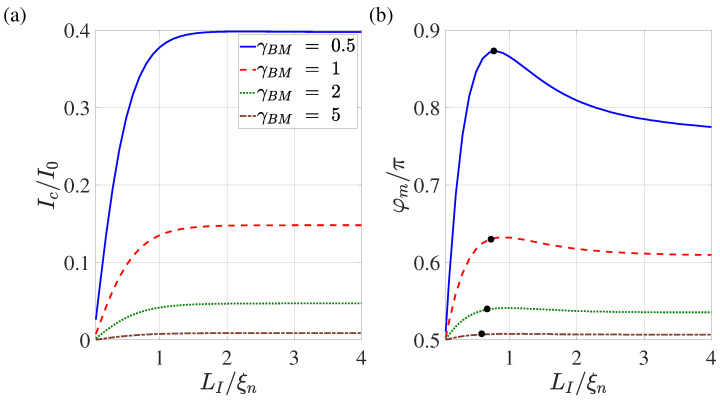
Dependence of the critical current (**a**) and the corresponding global phase (**b**) on the overlap length $L_{I}$ calculated for $\gamma_{BM}=0.5,1,2,5$. Other parameters: $T/T_{c}=0.5$, $d_{n}/\xi_{n}=0.1$, $d_{s}/\xi_{n}=10$, L=20, $\rho_{s}/\rho_{n}=10$, $L_{b}/\xi_{n}=0.1$. The black dots on the curves indicate the values of functions obtained at a distance removed from $x=L_{b}/2$ by an amount equal to the effective coherence length $\xi_{eff}$ at ω=πT.

Figure 9 shows the dependence of the critical current $I_{c}$ and the global critical phase $\varphi_{m}$ on the overlap length $L_{I}$ calculated for different values of the suppression parameters $\gamma_{BM}$. The points on the curves in Figure 9b indicate positions in the N film that are spaced from $x=L_{b}$ by the characteristic length $\xi_{eff}$ (3) calculated for ω=πT.

It can be seen that with $L_{I}/\xi_{n}≳2$ both of these parameters are weakly dependent on $L_{I}$. With such a noticeable overlap of the S and N films, part of the current has the ability to flow through the N film for a sufficiently long distance before leaking into the S electrode in a vicinity of $x≲L_{b}/2+L_{I}$. This decreases the current concentration in the S film in the vicinity of $x≳L_{b}/2$. At $L_{I}/\xi \approx$ 2, these two areas of current leakage overlap. With a further decrease in $L_{I}$, the current concentration region in the S-electrode is compressed and the additional contribution to linear inductance increases. This leads to an increase in $\varphi_{m}$. At $L_{I}≲\xi_{n}$, the mechanisms of suppression of superconductivity by current injected into the N region are most pronounced. This is accompanied by a decrease in the critical current; consequently, $\varphi_{m}$.

**Figure 10:**
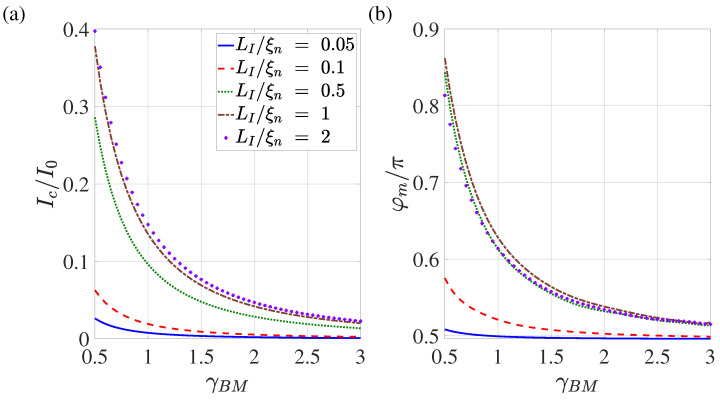
Dependence of the critical current (**a**) and the corresponding global phase (**b**) on the suppression parameter $\gamma_{BM}$ calculated for $L_{I}/\xi_{n}=0.05,0.1,0.5,1,2$. Other parameters: $T/T_{c}=0.5$, $d_{n}/\xi_{n}=0.1$, $d_{s}/\xi_{n}=10$, L=20, $\rho_{s}/\rho_{n}=10$, $L_{b}/\xi_{n}=0.1$.

Figure 10 shows the dependencies of the critical current and $\varphi_{m}$ on the suppression parameter $\gamma_{BM}$ for various overlap lengths $L_{I}$. In full accordance with the results presented in Figure 9, it can be seen that with the growth of $L_{I}$ the dependencies of $Ic(\gamma_{BM})$ and $\varphi_{m}\left(\gamma_{BM}\right)$ appear out on the universal curves, demonstrating the independence of these parameters from $L_{I}$.

## 7. Discusion

The performed study of electronic transport in planar Josephson SN-N-NS devices clearly demonstrates that the spatial redistribution of the supercurrent in the electrodes results in delocalization of their weak link region and a significant change in the shape of current–phase relation in these structures.

Our predictions can be verified experimentally by direct measurements of the current–phase relationship of these structures as well as by experimental study of the predicted dependence $I_{c}\left(L_{I}\right)$ shown in Figure 9.

We have shown that as the length of the $L_{I}$ of the SN interface decreases, the weakest place in the weak link region moves from the N metal to the SN boundaries. In fact, as shown in Figure 9, for values of $L_{I}$ greater than $\xi_{eff}$, the critical current of the structure does not depend on $L_{I}$. As $L_{I}$ decreases, the current density injected into the S electrode not only increases, it becomes more spatially homogeneous, meaning that $I_{c}$ becomes directly proportional to $L_{I}$. It is important to note that the degree of symmetry of such a double-barrier structure is determined by the difference in the length of the SN boundaries, and not by the difference in their transparency coefficients.

In the experimental study of structures in which a topological insulator plays the role of a normal metal, this remark is particularly important. The decay length in ballistic channels carrying superconducting current in topological materials can significantly exceed the $\xi_{n}$ of dirty films of normal metals. Because of this, the $L_{I}\approx \xi_{eff}$ ratio can be easily realised in a real experimental situation ($L_{I}≳100$ nm). In this case, the subject of experimental research is not the transport properties of a topological insulator; rather, it is the properties of its least-extended boundary with a superconducting electrode. As the transparency of the SN boundary is not much different from unity, the temperature dependence of the critical current of such a structure does not differ markedly from that predicted for pure ballistic contacts. Therefore, the fact that the experimental dependence $I_{c}\left(T\right)$ coincides with the theoretical dependence for purely ballistic contacts does not provide a clear indication as to which of the structures (SNS or SINIS) was actually studied. Thus, in [45], the presence of two critical currents was detected in the structures obtained by sputtering of Nb on top of Fe-doped BiSbTe_2_Se flake. According to the authors, the existence of the second critical current results from the intrinsic superconductivity of the Nb–Fe-doped BiSbTe_2_Se interface. On the basis of the conclusions obtained from this work, the fact of the existence of two critical currents can be interpreted in a different way. If the Nb–Fe-doped BiSbTe_2_Se–Nb contact is not an SNS but an asymmetric SINIS structure, then its critical current is determined by the least extended SN boundary, and the second critical current (a voltage jump on the I-V curve at a finite value of a bias current) occurs as a result of the transition to the normal state of the second-most extended SN interface. From our point of view, the experimental determination of the dependence $I_{c}\left(L_{I}\right)$ should be an important first step in the study of the parameters of SN-N-NS structures, as it permits experimental evaluation of such important parameters as $\xi_{ef}$ as well as understanding the type of Josephson contact (SNS or SINIS) realized by the geometry of the SN-N-NS contact selected for subsequent experimental studies.

It should be noted that the predicted deformation of the shape of current–phase relationship should be present in the SN-N-NS contacts studied here as well as in any Josephson structure where there is a concentration or other redistribution of the superconducting current in the regions of the S electrodes bordering the weak coupling region, for example, in Dayem and variable thickness bridges.

In Dayem bridges, the effective cross-section of the area where the current is concentrated in the S electrodes decreases due to the narrowing of the width of this area [83]. In variable thickness bridges, the current is concentrated in both the thickness and width of the S electrodes. As in the case of SN-N-NS bridges, the current concentration in DB and BVT structures should be accompanied by an increase in the local values of the inductance of the electrodes per unit of their length, i.e., their significant deviation from the linear inductance of S films away from the constriction. As a consequence, this should lead to the experimental shape of the current phase relation of SN-N-NS, BVT, and DB structures determined using both rf and dc SQUIDs providing $I(\varphi_{g})$ and not I(φ). The fact that the CPR experimentally obtained in Dayem bridges [15,23,24,27,29,35,84,85] has a shape that coincides with that shown in Figure 8 indirectly confirms the correctness of our results. We emphasise again that in addition to containing information about the contacts themselves, the current phase dependencies obtained experimentally in these works depend on the structure of the current redistribution in their electrodes.

## 8. Conclusions

The approach we have demonstrated in this paper allows calculation of the parameters of the equivalent scheme of SN-N-NS, BVT, and DB structures. Their representation in the form of a serial connection of the Josephson contact having $I(\varphi_{g})$ and the linear inductance of conductive electrodes is useful for the interpretation of experimental data in bridges [13,86], analysis of processes in nano-SQUIDs [1,2,3,4,5,6,7,8,9,10,11], experimental determination of inductance in various low-current superconducting devices [3,9], and in the design of digital circuits [12,13,14].

The data presented in Figure 8, Figure 9 and Figure 10 allow us to conclude that the conclusions formulated in [47] about the prospects of using SN-N-NS contacts as control elements of superconducting digital devices are correct if the following conditions are met: the overlapping area of S and N films $L_{I}$ should exceed $2\xi_{n}$; and the suppression parameter $\gamma_{BM}$ should be of the order of one. In this case, the characteristic size of the contact $L_{g}$ is approximately equal to $L_{b}+4\xi_{n}$, and the shape of the dependence $I(\varphi_{g})$ does not differ much from the sinusoidal one. It is important to note that the numerical results presented in this paper should only be considered as orders of magnitude estimates. Exact values can be obtained by generalising the model to a 3D case, which will be the subject of future work.

Our calculations focused on structures with electrodes made of conventional superconducting materials, where the dirty limit conditions are usually met and the isotropic potential of superconducting electron pairing occurs. In contrast, high-temperature superconducting materials are pure metals; their pairing potential is anisotropic, and has a d-wave or p-wave type symmetry.

This difference is significantly manifested at the boundaries of materials with weak coupling regions, leading to the possible generation of subdominant order parameters and spontaneous currents [87,88,89,90,91,92]. However, these effects should mainly affect the magnitude of the critical current of the structures. The concentration of the current in the electrode region bordering on the concentrating current weak link occurs independently of the differences formulated above. This means that delocalisation of the weak coupling region and the associated changes in the shape of the current–phase relationship are universal phenomena which should take place regardless of whether conventional or high-temperature superconductors are used as electrodes. Finally, it is important to note that there are geometries that result in current being concentrated in the electrodes at boundary surfaces with weak coupling.

## Data Availability

The data presented in this study are available on request from the corresponding author.
